# Isolation of suppressor genes that restore retrovirus susceptibility to a virus-resistant cell line

**DOI:** 10.1186/1742-4690-1-30

**Published:** 2004-09-28

**Authors:** Guangxia Gao, Stephen P Goff

**Affiliations:** 1 Department of Biochemistry and Molecular Biophysics Howard Hughes Medical Institute Columbia University College of Physicians and Surgeons New York NY 10032, USA; 2 Institute of Microbiology, Chinese Academy of Sciences, Beijing 100080, China

## Abstract

**Background:**

Genetic selections in mammalian cell lines have recently been developed for the isolation of mutant cells that are refractory to infection by retroviruses. These selections have been used to recover lines that block early postentry stages of infection, either before reverse transcription or before nuclear entry. The mechanisms of action of these blocks remain unknown.

**Results:**

We have devised a method for the selection of genes from cDNA libraries that suppress the block to virus infection, and so restore virus susceptibility. The protocol involves the transformation of pools of resistant cells by cDNA expression libraries, followed by the selection for rare virus-sensitive cells, using multiple rounds of selection after infection by marked viral vector genomes. The suppressor genes were then recovered from these virus sensitive cells, and their ability to restore virus susceptibility was confirmed by reintroduction of these cDNAs into the resistant line.

**Conclusions:**

The identities of these genes provide insights into the mechanism of virus resistance and will help to define new pathways used during retrovirus infection. The methods for gene isolation developed here will also permit the identification of similar suppressors that modify or override other recently identified virus resistance genes.

## Background

It is becoming increasingly apparent that mammalian cells harbor numerous genes that induce intracellular blocks to retrovirus infection [1,2]. These genes have presumably evolved and been maintained in the genome in response to the pathogenic and lethal consequences of infection, and are now thought to constitute an important part of the host defense against these viruses. Some of the genes and gene products responsible for this resistance have been recently identified, including the Fv1 locus in the mouse, which blocks infection after reverse transcription but before nuclear entry and establishment of the integrated provirus [3]; the APOBEC3G enzyme, which is incorporated into virion particles and catalyzes the destructive deamination of the viral cDNA during reverse transcription [4]; and the TRIM5a protein, which somehow blocks incoming virus soon after entry and prevents the activation of reverse transcription [5]. Others likely remain to be identified.

We have been involved in the development of screens and selections for virus resistance genes, and have isolated mutant cell lines after chemical mutagenesis that are profoundly resistant to retrovirus infection. Two such lines isolated from a parental fibroblast cell line, Rat2 cells, have been characterized in some detail [6]. Mutant line R3-2 exhibited a nearly 1000-fold resistance to infection by genetically marked Moloney murine leukemia virus genomes, and was resistant to pseudotyped viruses utilizing the ecotropic envelope, the amphotropic envelope, or even the VSV G envelope protein. Infection of R3-2 resulted in the normal synthesis of the linear viral DNA by reverse transcription, but circular viral DNAs and integrated proviruses were not generated. The viral DNA was apparently trapped in the cytoplasm in a form that was not readily extracted by conditions that allowed DNA recovery from wild-type infected cells. Mutant line R4-7 exhibited about a 100-fold resistance to infection by M-MuLV, also independent of the envelope mediating entry. Infection of this line was blocked earlier, before the initiation of reverse transcription. Both lines R3-2 and R4-7 were also resistant to infection by pseudotyped HIV-1 viral vectors.

To probe the nature of the blocks in these mutant cell lines, we have sought to identify and characterize suppressor genes that override the restriction exhibited by these cells. To identify such genes, we have developed methodologies that allow for the selection of rare virus-sensitive clones arising after transfer of gene libraries into populations of virus-resistant parents. We here report the isolation of two cDNA constructs that each restore virus sensitivity to the R4-7 mutant cell line. These DNAs constitute valuable tools in the characterization of this line's virus resistance.

## Results

### Selection for virus-sensitive clones from R4-7 mutant cells expressing cDNAs

**Figure 1:**
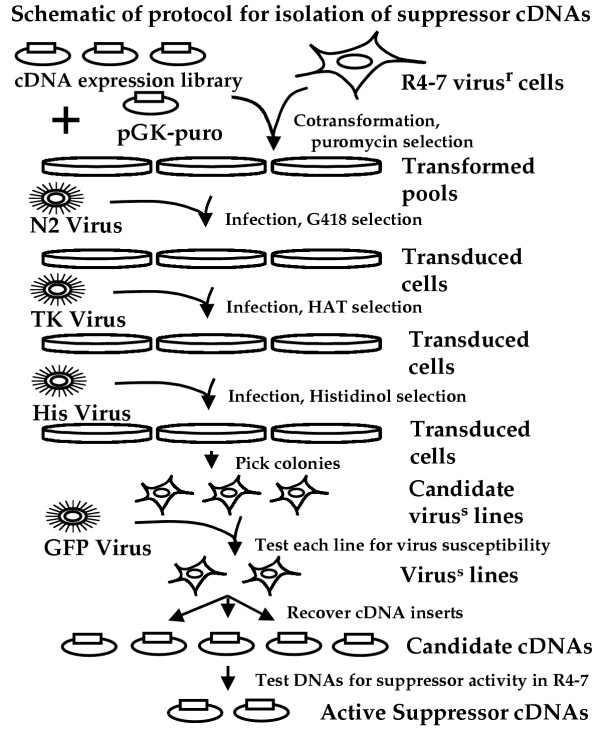
Flowchart for isolation of cDNAs that suppress virus resistance and restore virus sensitivity to the R4-7 mutant cell line. See text for description.

The R4-7 mutant cell line is approximately 100-fold resistant to transduction by MuLV-based vectors as compared to wild-type Rat2 cells [6]. To identify genes that could suppress this phenotype and restore virus sensitivity, a protocol involving multiple rounds of selection for virus sensitivity was devised (Fig. 1). First, R4-7 cells were transformed by a library of rat kidney cDNAs expressed from the constitutive CMV promoter. Recipient cells were selected by cotransformation with a DNA expressing puromycin resistance. Five pools of the puromycin-resistant cells were generated and maintained separately, each pool containing more than 1000 independent transformed clones. The expectation was that multiple rounds of selection for virus-sensitive clones would be required to recover such cells, with each round providing at most a 100-fold enrichment.

Four of the pools of transformed cells, with each clone in the pools overexpressing a small number of cDNAs, were sequentially exposed to a series of three genetically marked ecotropic MuLV-based vectors, and the rare successfully infected cells were recovered after each infection by selection for the marker carried by the vector (see Methods). The cells were first exposed to N2 virus, an MuLV vector carrying the neor marker, and infected cells were selected in medium containing G418. These cultures were then expanded and exposed to Eco-TK virus, an MuLV vector carrying the Herpes virus TK gene, and infected cells were selected with HAT medium. These cultures were expanded and finally exposed to Eco-His virus, and infected cells were selected with medium containing histidinol. In all cases, the selecting viral vectors were applied at low multiplicities of infection (MOI) so as not to override the resistance of the parental R4-7 cells, as can happen at high MOI [6]. Individual colonies were recovered after the triple selection.

The number of colonies of infected cells recovered at each stage of the selection was determined for each of the four pools (Table 1). The number of colonies of wild-type Rat2 cells exposed to the virus in parallel was determined for comparison. In each of the first two rounds of selection, the pools of mutant cells yielded about 25-fold fewer transductants than the wild-type control, indicating retention of the resistance in the bulk of the population. In the third round, pools 3 and 4 yielded slightly higher numbers of colonies than the other pools, suggesting possible enrichment for virus sensitive clones, though still less than the wild-type cells. A total of 36 candidate colonies were isolated.

**Table 1:** Numbers of colonies recovered after each round of infection and selection

	Initial Cell Population				
	Pool 1	Pool 2	Pool 3	Pool 4	Rat2

Puro^R ^colonies after transfection	>1000	>1000	>1000	>1000	-
Neo^R ^colonies after N2 virus infection	~400	~400	~400	~400	TMTC
HAT^R ^colonies after TK virus infection	100	60	40	30	2000
His^R ^colonies after His virus infection	3	2	12	19	~200
Virus^S ^lines by GFP virus susceptibility	0	0	2	4	-

To determine whether any of these candidate clones had become truly virus sensitive, all 36 colonies were individually picked and expanded into larger cultures. These cultures were then tested by infection with Eco-GFP, a virus vector expressing the green fluorescent protein, and the fraction of the cells expressing the marker was determined by inspection. While all the clones from pools 1 and 2 were as resistant as the parental R4-7 line, a total of 6 clones – 2 from pool 3 (dubbed A1, A2) and 4 from pool 4 (dubbed B1, B2, C1, C2) – were fully sensitive to infection. These cloned lines were thus candidates as potentially carrying cDNAs that could restore virus sensitivity to the R4-7 line.

### Recovery of cDNAs from virus-sensitive cell lines capable of suppressing virus resistance

To recover the cDNAs present in the virus sensitive cell lines, total genomic DNA was isolated, and polymerase chain reactions were performed to amplify expression cassettes composed of the CMV promoter, the cDNA insert of the library and the poly(A) addition signal. The amplified DNA from each line was directly cloned into the TOPO plasmid DNA and used to transform bacteria. In this way cloned cDNAs were recovered from five of the six lines. Because the lines were expected to each carry a few different cDNAs, and because only one cDNA in each line would be expected to be responsible for the phenotype, a total of 50 bacterial colonies were isolated for each of the five lines. DNAs were prepared from these bacterial colonies and assigned to groups based on the pattern of restriction fragments after produced after digestion with MspI. The number of distinct cDNAs recovered from each of the five lines ranged from 1 to 11, and all together included 29 cDNAs (Table 2).

**Table 2:** Numbers of cDNAs recovered from Virus^S ^cell lines

	Origin of cell lines						Total
	Pool 3	Pool 3	Pool 4	Pool 4	Pool 4	Pool 4	

Virus^S ^cell line	A1	A2	B1	B2	C1	C2	
Total cDNAs examined	50	50	50	50	50	-	250
Distinct cDNAs	10	2	5	1	11	0	29
Active cDNAs	0	0	1	0	1	0	2

**Figure 2:**
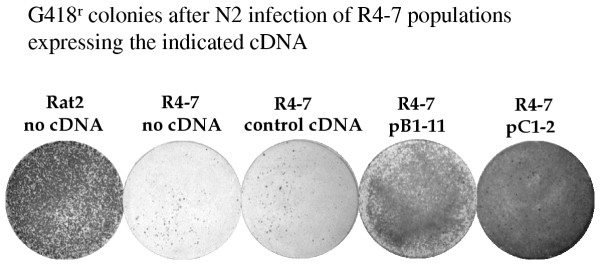
Ability of the suppressor cDNAs to restore virus susceptibility to the R4-7 mutant cell line. R4-7 cells were cotransformed with the indicated cDNAs, and the transformants were pooled and grown into cell populations. These cultures were then exposed to equal amounts (approximately 10,000 cfu in NIH/3T3 cells) of an N2 virus preparation, and virus susceptibility was assessed by plating the infected cells in medium containing G418. While the mutant R4-7 control populations yielded only ~50 colonies, the populations expressing the active cDNAs produced nearly confluent lawns. Rat2: virus-sensitive subclone isolated after mutagenesis. R4-7: mutant line. No cDNA: pGKpuro marker DNA alone. Control cDNA: marker plus inactive cDNA. PB1-11, pC1-2: marker plus indicated cDNA.

**Figure 3:**
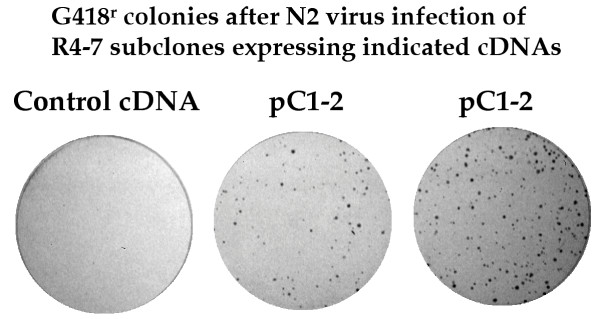
Restoration of virus susceptibility by pC1-2 DNA in clonal cell lines. Single-cell clones were derived from R4-7 cell populations cotransformed with either inactive control cDNA or pC1-2 DNA. The resulting lines were exposed to N2 virus (approximately 300 cfu in NIH/3T3 cells) and plated in medium containing G418. While the control yielded no colonies, the clonal lines containing pC1-2 showed 100–200 colonies.

The cDNAs isolated from the virus sensitive lines were then tested directly for their ability to suppress the virus resistance of R4-7 cells. Each cDNA (20 ug) was mixed with pGK-puro DNA (2 ug) and used to transform naive R4-7 cells, and recipients stably expressing the DNAs were selected by growth in puromycin. The resulting puromycin-resistant colonies derived from a given cDNA were pooled and grown into large cultures, and the resulting populations were tested for sensitivity to Eco-neo virus infection. Two of the cDNAs, one from cell line B1 (designated pB1-11) and one from cell line C1 (designated pC1-2), dramatically suppressed the virus resistance of R4-7 cells (Fig. 2). An inactive cDNA retained as a negative control did not suppress the resistance. The susceptibility to infection of the pooled R4-7 transfectants for the two active clones was similar to that of the wild-type Rat2 cells, and roughly 100-fold higher than that of the R4-7 parents. To further document the sensitivity of induced by pC1-2, two individual clones were isolated from the R4-7 populations expressing pC1-2 and a control cDNA, and these clones were similarly tested by infection with Eco-neo virus. Like the pooled populations, the clones expressing pC1-2 were virus-sensitive and the controls were not (Fig. 3). Thus, these cDNAs were sufficient to suppress the resistance, and were likely responsible for the virus sensitivity of the two lines in which they were recovered after the triple selection. The remaining lines had presumably become sensitive to virus independently of any of the cDNAs they carried, or as a result of a cDNA that was not recovered from the PCR amplified DNA products.

### Characterization of biologically active suppressor cDNAs

**Figure 4:**
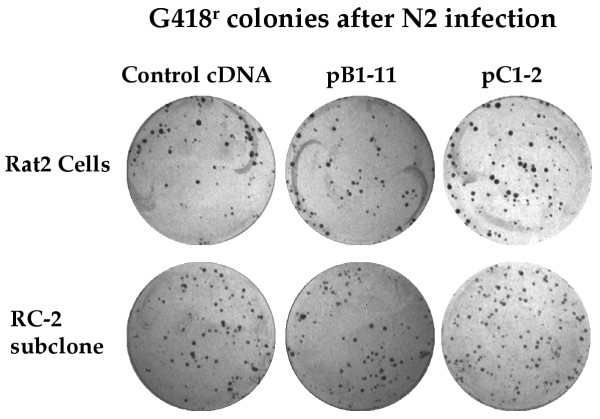
Lack of effect of suppressor cDNAs in wild-type cells. Rat2 or a virus-sensitive subclone isolated after mutagenesis (RC-2) were cotransformed with the indicated DNAs, and the transformants were pooled and grown into cell populations. The resulting cultures were exposed to N2 virus (approximately 300 cfu in NIH/3T3 cells) and plated in medium containing G418. All the cultures yielded approximately equal numbers (~200) of colonies.

The pB1-11 and pC1-2 DNAs could function as general enhancers of retrovirus infection, or alternatively as specific suppressors of the block in the R4-7 mutant cell line. To distinguish between these possibilities, the DNAs were introduced into the wild-type Rat2 cells, the distinct R3-2 mutant line, and the R4-7 line by cotransformation, and stable transformants were selected and expanded. The resulting transformed lines were then tested for their sensitivity to infection by Eco-Neo virus. The Rat 2 lines expressing pB1-11 showed no change in virus susceptibility, and the Rat2 lines expressing pC1-2 showed at most a 2-fold increase in sensitivity (Fig. 4). The corresponding R3-2 lines gave similar results (data not shown). Thus, both cDNAs were highly specific in enhancing the virus susceptibility of the R4-7 line.

The DNA sequences of the two cDNAs were determined and compared with the nucleic acid sequences of the NCBI databases. Clone pB1-11 contained an insert of 855 bp with close sequence similarity to the central portion of a transcript originally termed HCC1.3/1.4, identified as encoding a prominent autoantigen expressed in a human hepatocarcinoma [7]. The similar mouse gene product, dubbed CAPER, was subsequently shown to interact with c-Jun, a subunit of the AP-1 activator, and the estrogen receptors ERa and ERß, and to exhibit transcriptional coactivator activity when expressed in concert with these transcription factors [8]. The cDNA insert of pB1-11 aligned well with both the human sequences (92% identity match to bp 910–1767 of HCC1.4 (Genbank accession no. L10911)) and the mouse sequences (94% identity match to bp 1153–2006 of CAPER (accession no. AY061882)). Remarkably, the cDNA fragment was inserted in reverse orientation relative to the CMV promoter of the pcDNAI plasmid vector [9], and thus the active DNA would produce an antisense mRNA transcript.

**Figure 5:**
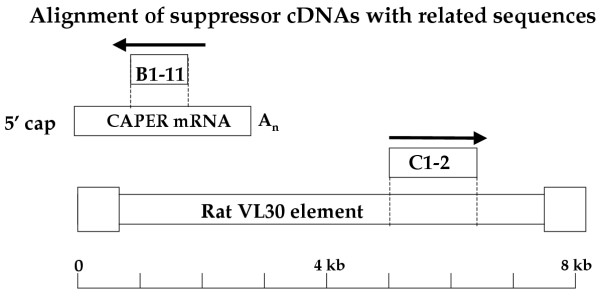
Sequence alignment of pB1-11 and pC1-2 DNA inserts with similar sequences from NCBI database. The insert of pB1-11 (855 bp) is an antisense sequence match to the central portion of the CAPER mRNA [8], and that of pC1-2 (1407 bp) is a sense sequence match to a portion of the VL30 endogenous retrovirus-like element [17].

Clone pC1-2 proved to contain an insert of 1407 bp, with close sequence similarity to a central portion of the VL30 elements, a family of endogenous retrovirus-like elements widely expressed in many mouse [10-14] and Rat cell lines [15,16]. The pC1-2 sequences aligned best with particular Rat elements expressed in tumor cells (~88% identity to bp 5025–6151 of a 7.4-kb element [17]; Genbank accession no. D90005) and in the ovary (~90% identity to bp 3341–4677 of a 5.5-kb element [18]; Genbank accession no. U48828). There was weaker similarity to related retroviruses, such as the gibbon ape leukemia virus [19]. The insert was in the sense orientation relative to the CMV promoter, and if transcribed would result in formation of a plus strand RNA, corresponding to the central portion of the VL30 transcripts. Like most rat VL30 elements, the insert did not include any significant open reading frames, but rather contained numerous mutations that introduced frameshifts and stop codons that would preclude synthesis of any long protein products. These results suggest that both of the pB1-11 and pC1-2 DNAs might function by virtue of their RNA products rather than any encoded proteins. The sequences of the two inserts have been submitted to the NCBI database (pB1-11 accession number is AY769432; pC 1-2 accession number is AY769433; see figure 5).

### Expression of CAPER and VL30 RNAs in R4-7 mutant line

**Figure 6:**
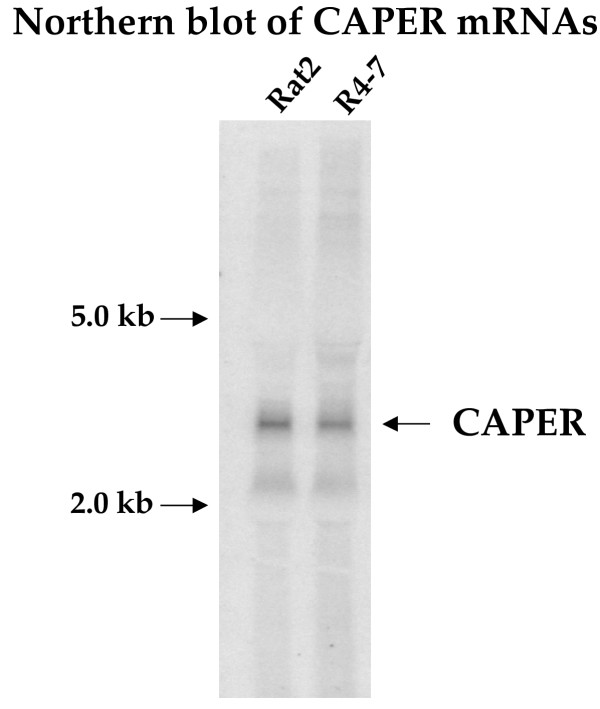
Northern blot analysis of mRNAs in parental Rat2 and mutant R4-7 cell lines. RNA preparations from the indicated cells were separated, blotted, and hybridized with a 32P-labeled pB1-11 probe. The major mRNA at ~3.0 kb is indicated. The position of the 28S and 18S rRNA markers are indicated on the left.

The biological activity of the pB1-11 and pC1-2 DNAs in restoring virus susceptibility could be mediated through effects on their corresponding endogenous gene products expressed in the R4-7 mutant cell line, or could be indirect. If their activity was direct, then either one of the corresponding endogenous genes – the CAPER gene or a VL30 element – might be the locus that was originally mutated to give rise to the resistance of the R4-7 line. To examine this possibility, RNAs were prepared from R4-7 and wild-type cells, and analyzed by Northern blot. Hybridizing with the pB1-11 probe showed a single major RNA about 3 kb in length in both lines, with no significant change in level detected (Fig. 6). Hybridization with the pC1-2 probe showed an intense smear of RNAs in both lines as typically seen for VL30 RNAs (data not shown). No differences between the lines was apparent.

Although the levels of the CAPER mRNA was not detectably altered in the R4-7 line, it remained possible that the gene and its transcripts harbored point mutations that were responsible for the virus resistance. To test this possibility, CAPER cDNAs were isolated from the R4-7 and Rat2 cells by RT-PCR, and the amplified sequences were cloned into the TOPO vector. Ten cDNA clones from each line were recovered and sequenced. Clones of three distinct structures were recovered from each line, likely arising by alternative splicing, but the sequences of the corresponding clones from the two lines were identical (data not shown). These results suggest that the CAPER gene is likely not mutated in the R4-7 line. Nevertheless, to test whether any of these cDNAs could alter virus susceptibility, the cDNA inserts from both R4-7 and Rat2 cells were transferred into the expression vector pcDNA3.1/zeo (see Methods). Overexpression of the various CAPER cDNAs from the Rat2 cells in R4-7 cells did not restore virus susceptibility, and overexpression of the corresponding cDNAs from the R4-7 cells did not induce virus resistance. Thus, of the various CAPER expression constructs, only the original pB1-11 antisense DNA had biological activity.

## Discussion

The results here document the development of an effective procedure for the isolation of cDNAs that allow virus infection of virus-resistant cells. The key feature is the repeated infection of the parental resistant line with viral vectors carrying distinct selectable markers, and has become possible only with the development of a multitude of such markers. In this way, rare susceptible cells in the R4-7 population are enriched by as much as 10 to 100 fold in each round of selection. The protocol should allow the recovery of DNAs that confer susceptibility from any large library, if present at an abundance of perhaps at least one in 106 clones. The system can only work if a single DNA is sufficient to enhance virus susceptibility. Once cell lines with restored virus sensitivity were isolated, the recovery of the cDNAs from genomic DNA and the screening for active clones were relatively straightforward.

The identities of the two sequences in the active cDNAs isolated here were surprising and the mechanisms of action of the two distinct clones remains mysterious. Both are highly potent, restoring virus susceptibility essentially to wild-type levels (figure 2). The time of the block to replication in the R4-7 line that is overcome by these two DNAs is very early after virus entry, before the initiation of reverse transcription by the incoming virus [6]. One possibility is that the mutant line fails to uncoat the virions sufficiently to allow deoxyribonucleotides into the core. In this scenario the cDNAs would somehow facilitate the uncoating process or inhibit a block to uncoating.

HCC1.3 and HCC1.4 are two closely related cDNAs first recovered from a patient with hepatocellular carcinoma [7]. The encoded protein was a prominent nuclear autoantigen. The deduced amino acid sequences contain an arginine/serine rich domain and three ribonucleoprotein consensus sequence domains, often found in RNA splicing factors; they show weak homology to *S. pombe *GAR2, a nuclear protein. A later report demonstrated that the gene product interacted with the transcriptional activators AP-1 and the estrogen receptors ERa and ERß, and had potent cotransactivation activity; the gene was renamed CAPER, for coactivator of AP-1 and ER [8]. The antisense orientation of the pB1-11 cDNA suggests that its mechanism of action might be to lower the level of the endogenous sense mRNAs and the encoded proteins produced from the CAPER gene. We were unable to directly assess the level of the mRNAs in the presence of the antisense cDNA by Northern blots because the level of expression of the antisense RNA was so much higher than the endogenous mRNA that these transcripts were obscured. However, it is possible that the reduction in levels of a protein factor involved in regulation of transcription could elicit profound changes in the patterns of gene expression in the cell. We cannot rule out the remote possibility that a cryptic promoter results in some production of sense mRNA, and a protein fragment with biological activity, from the pB1-11 cDNA.

Whatever the mechanism of action of the pB1-11 cDNA, it is unlikely that the endogenous CAPER gene is the locus of the original virus resistance mutation in the R4-7 line. The levels of the major mRNA are similar in the mutant and the wild-type parent (Fig. 6), and sequence analysis of a variety of cDNAs from parent and mutant lines did not uncover any mutations. Further, the overexpression of the CAPER cDNAs from R4-7 did not cause resistance, and the overexpression of the wild-type CAPER cDNA did not suppress the resistance. Rather, the antisense cDNA must correct the phenotype indirectly, likely through effects on gene expression. The localization of the HCC1.3/1.4 or CAPER protein in the nucleus [7] rather than at the site of virus arrest also suggests that its mechanism is indirect. Possibly CAPER acts to maintain a program of cytoplasmic protein expression that blocks virus infection in the R4-7 mutant line.

The VL30 elements are a very large family of endogenous virus-like genes found in both mouse and rat genomes [10-14]. The various elements are dispersed and have significantly divergent sequences. Though *gag*- and *pol*-related sequences are often recognizable, nearly all the elements are grossly defective, with multiple frameshift and premature termination mutations interrupting the open reading frames. In addition, the majority of the elements have suffered deletions of various regions relative to the longer family members. Thus, while many of the elements are highly transcribed in rodent cell lines, very few of the transcripts code for protein products of significant length. However, the VL30 RNAs often contain recognition elements for packaging into virion particles encoded by murine leukemia viruses, signals for initiation of DNA synthesis and strong stop DNA translocation, and termini recognized by viral integrase proteins, and thus are competent for transfer by replication-competent viruses acting as helpers.

The sequence of the insert in pC1-2 corresponds to a portion of the retroviral *pol *gene, specifically the integrase coding region, but is typical of the VL30s in containing no long ORF; furthermore, known *cis*-acting regions needed for replication are absent. We suppose that the RNA itself may be responsible for the activity, perhaps by binding some cellular protein. The region of the VL30 genome present in pC1-2 – the 3' portion of the *pol *gene – is not known to contain a binding site for any particular protein. The corresponding region of replication-competent viruses, however, would normally contain the splice acceptor site for the envelope mRNA. Although many VL30 elements do not contain *env *genes, and although the splice acceptor sites are not readily apparent in the pC1-2 sequence, the transcript might be hypothesized to bind splicing machinery. In this scenario, a possible mechanism of action of the clone is for the overexpressed RNA to bind up a splicing factor or other RNA binding protein, titering out the free protein and removing it from solution. If this factor were responsible for the viral resistance of the R4-7, either directly or indirectly, the binding might relieve that block. The mechanism would be surprising only because the endogenous VL30 RNAs are already so abundant in Rat2 cells, and they are not able to suppress the block to infection. However, the pC1-2 sequence must be an unusual element, in that some distinctive aspect of its sequence must be responsible for its peculiar biological activity. Perhaps identifying proteins that bind to the pC1-2 transcript would be informative.

The mechanism of resistance exhibited by the R4-7 line remains uncertain. The block is early but likely not at virus entry: it occurs whether ecotropic envelope, amphotropic envelope, or even the VSV G protein is used for entry [6]. Further, the block is unlikely to involve VSV G function, since the cells are susceptible to infection by VSV itself (J.-W. Carroll and M. MacDonald, Rockefeller University, unpublished observation). The early block occurs at a similar stage of infection – before reverse transcription – as the dominant block induced by TRIM5a, a gene responsible for retrovirus resistance in primates [5]. There are no other indications, however, that the two blocks are related. We have observed that the R4-7 cells exhibit a slightly different morphology than the parental Rat2 cells, being somewhat more rounded and more easily detached from the substrate during trypsinization. This phenotype could in principle be unrelated to the virus resistance, since the cells were subjected to heavy chemical mutagenesis before their isolation [6]. However, the R4-7 cells expressing both pB1-11 and pC1-2 were restored to a flatter morphology, much closer to that of the parental line, suggesting that the two phenotypes may be causally linked. If this notion is correct, changes in the cytoskeleton may be involved in the resistance. Further analysis of the R4-7 cells by gene expression profiling may help reveal the basis for its behaviors.

## Conclusions

The power of genetic selections in mammalian cells for alterations in virus susceptibility is increasing rapidly. We believe that selections like the one devised here will be applicable to the isolation of suppressors of other blocks to infection, including the prototypical Fv1 gene [3], the APOBEC3G cytosine deaminase [4], and the TRIM5a gene [5]. The identity of such suppressors may provide important clues into the mechanism of their action and regulation.

## Methods

### Cell lines, cell culture

The Rat2 cell line is a TK-negative fibroblast line that is highly sensitive to MuLV infection. RC-2 is a subclone isolated after mutagen exposure but also sensitive to virus, and was used in many experiments as a wild-type control line. Lines R3-2 and R4-7 are virus-resistant mutants of Rat2 isolated after exposure to ICR-191 [6]. 293T cells are human embryonic kidney cells transformed by adenovirus E1 and also expressing SV40 T antigen. All these lines were maintained in DMEM with 10% fetal calf serum.

### DNA transformations

A rat kidney cDNA library in the pcDNAI vector [9] was purchased from Invitrogen (Carlsbad, CA). To increase the library transfection efficiency and maximize the integrity of the cDNAs, the library was digested in the vector sequence with the restriction enzyme SfiI and then religated. R4-7 cells in ten 10-cm dishes were cotransformed with 20 ug of the religated library DNA and 2 ug of pGK-puro plasmid by calcium phosphate-mediated transformation. Cells expressing transformed DNAs were selected by growth in culture medium containing 5 ug/ml puromycin. Each transformed cell was expected to receive about 2–10 different cDNAs. The cultures were expanded before the selections for virus susceptibility such that a pool of 105 cells contained about 2000 distinct puromycin resistant clones. Thus, there were about 50 sibling cells of each transformant in the pools at the time of selection for virus susceptibility.

### Retrovirus preparations

Eco-neo or MuLV-N2 virus [20]; Eco-TK virus, and Eco-GFP virus [21] were as previously described [6]. To generate Eco-His virus, the Neo resistance gene in the N2 vector was replaced with His resistance gene, and GP+E86 packaging cells [22] were stably transformed with the resulting vector DNA. Recipients were selected with histidinol and the resistant cells were pooled to generate Eco-His producer cells. Typical titers of the virus preparations on Rat2 cells were 107 cfu/ml for N2 virus; 2 × 104 for TK virus; 2 × 106 for Eco-His virus; and 105 cfu/ml for Eco-GFP virus.

### Viral transduction and selection

Selections for virus sensitive cells were performed by infecting approximately 105 R4-7 cells per 10-cm dish in each round, with virus titers determined by infection of Rat2 cells. In the first round approximately 104 cfu of N2 virus were applied, and transductants were selected with 800 ug/ml G418. In the second round, approximately 2 × 103 cfu of Eco-TK virus were used, and transductants were selected with HAT medium (Gibco). In the third round, approximately 200 cfu of Eco-His virus were applied, and transductants were selected with medium containing 1 mg/ml histidinol. The multiplicities of all these infections with the selecting viruses were kept low, at less than 0.1 These low MOIs were required because infection of R4-7 cells at high MOI can override the block, perhaps by saturation of a titratable factor. Even at this low MOI, the presence of many siblings of each transformant implied that most cDNAs in the pool were tested for inducing virus susceptibility.

### Polymerase chain reactions

cDNA inserts from the expression library were recovered from cell lines by PCR as follows. Genomic DNA was extracted (DNAeasy kit, Qiagen) and subjected to PCR with primers hybridizing upstream from the CMV promoter (sequence 5'-GGGCCAGATATACGCGTT-3') and downstream from the poly(A) addition region (sequence 5'-AATTTGTGATGCTAT-3') of the pcDNAI vector. Conditions for the PCR were: ten cycles of 94°C for 10 sec, 55°C for 30 sec, and 68°C for 3 min, followed by 20 cycles of the same conditions but with an increase in the polymerase reaction time of 5 sec in each cycle. The amplified DNAs were cloned directly into the TOPO vector (Invitrogen) and used to transform DH10b bacteria to ampicillin resistance. DNAs were isolated from approximately fifty bacterial colonies for each original cell line.

CAPER cDNAs were prepared from RC-2 mRNA preparations by standard RT-PCR methods using primers spanning the entire ORF (sequences: 5'-ATATAGCTTAAGGCCACCATGGCAGACGATATTGATAT-3' and 5'-ATATAGGCGGCCGCTCATCGTCTACTTGGAAC-3'), and cloned into the pcDNA3.1/zeo expression plasmid using AflII and NotI restriction sites.

## Authors' contributions

GG carried out all the experiments and participated in their design. SPG participated in the experimental design and drafted the manuscript. Both authors read and approved the final manuscript.
